# Efficacy and safety profile of Glucagon-Like Peptide-1 Receptor Agonist in obese Type-2 diabetes patients from a private institution in Karachi

**DOI:** 10.12669/pjms.39.4.7353

**Published:** 2023

**Authors:** Saeeda Fouzia Qasim, Tasnim Ahsan, Saima Ghaus, Paras Imran

**Affiliations:** 1 Saeeda Fouzia Qasim, FCPS & MRCP (Ireland) Jinnah Postgraduate Medical Centre, Karachi, Pakistan; 2 Tasnim Ahsan, MRCP & FRCP Jinnah Postgraduate Medical Centre, Karachi, Pakistan; 3 Saima Ghaus, FCPS & MRCP (UK) Medicell Institute of Diabetes, Endocrinology and Metabolism (MIDEM), Karachi, Pakistan; 4 Paras Imran, FCPS Jinnah Postgraduate Medical Centre, Karachi, Pakistan

**Keywords:** Diabetes Mellitus, GLP-1RA, Obesity, Glycemic control, HbA1c

## Abstract

**Objective:**

To assess the effectiveness of GLP-1RAs in managing obese T2DM patients.

**Methods:**

This prospective cohort analysis was conducted at Medicell Institute of Diabetes, Karachi, Pakistan; from July 2019 to July 2021. A total of 97 obese individuals >16 years of age with T2DM and IGT were initially enrolled, and 81 patients who showed up for the follow-up were prescribed one of the three GLP-1RAs available in Pakistan.

**Results:**

Out of 81 patients who showed up for the follow-up visit, 43 had received Liraglutide, 25 were taking Dulaglutide, and 13 had been prescribed IDegLira supplemented with oral hypoglycemic medications ± insulin. The mean age of the enrolled patients was 49.21(12.44) years, and there was female predominance (55.6%). Overall, there was a significant weight and BMI reduction among the patients treated with either of the GLP-1RAs (*P<.01*). Furthermore, significant glycemic control was observed in all three groups after the treatment. The Dulaglutide group demonstrated a more significant reduction of HbA1c compared to Liraglutide group, which showed more pronounced weight and BMI reduction. Nevertheless, this class of medications was well-tolerated, with nausea being the most often reported side effect.

**Conclusion:**

GLP-1RAs showed favorable weight and HbA1c reduction among patients of all three treatment groups.

## INTRODUCTION

Diabetes mellitus is a long-term ubiquitous condition with immense public health connotations, with a wide array of complications. Referring to the International Diabetes Federation (IDF), the worldwide preponderance of diabetes in 2021 was estimated to be 10.5% and is projected to rise to 12.2% by 2045.^1^ According to the reports, 33 million Pakistanis had diabetes in 2021, i.e., a 70% increase from 2019.^2^

There is a persistent unmet need for innovative glucose-lowering medication offering long-term glycemic control without causing hypoglycemia, weight gain, or fluid retention, which are known adverse effects of several currently available low-cost glucose-lowering drugs.^3^ New possibilities in managing diabetes were made possible by a greater understanding of the fundamental pathophysiology involved in diabetes. In particular, glucagon-like peptide-1 receptor agonists (GLP-1RAs) have profoundly shifted the diabetes treatment paradigm. With advantages extending beyond glucose management, these medications constitute a novel strategy for treating diabetes, with beneficial effects on weight, cholesterol levels, blood pressure, nonalcoholic fatty liver disease/steatohepatitis, beta-cell activity, and insulin sensitivity.^4^

Currently, five approved GLP-1 receptor agonists are available, including Exenatide, Dulaglutide, Liraglutide, Lixisenatide, and Semaglutide. For the treatment of chronic weight management in adults with a BMI greater than 27 kg/m^2^, at least one weight-related comorbidity, such as T2D or hypertension, or BMI >30 kg/m^2^, Semaglutide and Liraglutide are approved. Each drug in this class has distinct pharmacodynamic, pharmacokinetic, and clinical characteristics. Patient preferences, potential side effects, and cost endorse GLP-1RA use. However, in patients with clinical atherosclerotic cardiovascular disease, it is suggested to use either Liraglutide, Semaglutide, or Dulaglutide, as their protective effects are evident for the situation.^4^ GLP-1RA appears to decrease overall mortality in people with diabetes and established CVD; once-weekly formulations may also improve patient adherence.^5^,^6^

Pakistan has only had one locally performed study published so far that examined the efficacy of Liraglutide.^7^ In Pakistan, Liraglutide first became accessible in 2016, followed by Dulaglutide in 2018, and finally, a combination of Liraglutide and Insulin Degludec in March 2020. This study sought to evaluate the safety and efficacy of all available GLP-1RAs among T2D obese patients.

## METHODS

**Fig.1:**
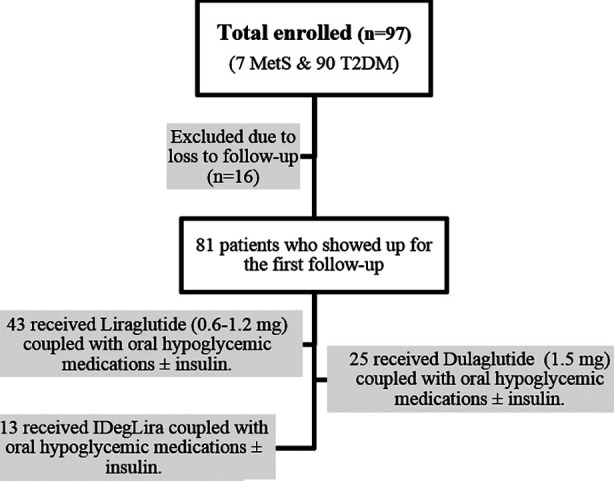
Study flow diagram.

This prospective cohort study was conducted between July 2020 and July 2021 at the Medicell Institute of Diabetes Endocrinology & Metabolism. The Institutional Review Board (Ref: No.F.2-81/2021-GENL/73459-A/JPMC, date Dec. 31, 2021) gave its prior approval, and informed consent was obtained from each patient. The sample size of 65 was calculated using WHO calculator for Sample size determination in Health Studies, considering 95% confidence interval, 5% absolute precision, and mean difference 1.48(0.83). In view of the attrition, the data of 97 patients were recorded, seven with metabolic syndrome and impaired glucose tolerance (IGT) and 90 patients with T2D (Fig.1). One of the three GLP-1RAs (available in Pakistan) was prescribed to 81 patients who showed up for the follow-up. The doses were modified in accordance with the glycemic levels.

### Inclusion & exclusion criteria:

Obese individuals > 16 years of age with T2D and IGT were included in the study. While patients with Type-1 diabetes, < 16 years of age, family history of medullary thyroid cancer, or a history of pancreatitis were excluded from the study sample.

### Assessments:

The data was collected using a pre-designed proforma; baseline demographics, comorbidities and primary outcome variables, such as weight, BMI, glycosylated hemoglobin (HbA1c), and secondary outcome variables including lipid profile, FBS, alanine aminotransferase (ALT), and cardiovascular parameters i.e., blood pressure, and heart rate were documented. The clinical, biochemical estimations and safety evaluation were performed at baseline and follow-up (six months after initial assessment).

### Statistical analysis:

Data were analyzed using SPSS version 23.0. All quantitative variables were displayed using mean and standard deviation. Within-group data with normally distributed characteristics were analyzed using paired sample t-test. For skewed data, we presented the non-parametric Wilcoxon Sign Rank test. One-way ANOVA was utilized to assess the baseline and follow-up comparisons (symmetric) between the treatment groups, while the Kruskal Wallis test was employed for asymmetric distributions. The Pearson Chi-Square test was used to see whether the reported comorbidities and side effects varied across the treatment groups. Statistical significance was defined at a p-value < 0.05.

## RESULTS

The comparison of baseline characteristics, variation in the biochemical estimations and outcome variables from baseline to follow-up visits among the patients treated with various GLP-1Ras are shown in Table-I. Metformin was used by 65 patients (80.2%), and sulphonylurea was taken by 18 patients (22.2%). Most patients had diabetes for over five years, and hyperlipidemia was present in 55.0% of the individuals. The overall changes in the biochemical estimations and outcome variables among T2D patients treated with GLP-1RAs is shown in Table-II. A significant reduction in the weight, HbA1c and cholesterol level was observed at the follow-up visit (p<.01).

**Table-I:** Comparison of baseline characteristics, outcome variables and other biochemical estimations among treatment groups.

Variables			Treatment Groups			p-value

			Dulaglutide (n=28)	Liraglutide (n=38)	IDegLira (n=15)	
Baseline Characteristics	Age; years		52.14(10.78)	46.67(14.01)	52.75(6.77)	0.139
	Gender	Female	12(48.0)	27(62.8)	6(46.2)	0.376
		Male	13(52.0)	16(37.2)	7(53.8)	
	Comorbidities	Hypertension	13(52.0)	23(53.5)	11(84.6)	0.105
		IHD	1(4.0)	3(7.0)	1(7.7)	0.859
		DL	16(64.0)	25(58.1)	12(92.3)	0.075
		RA	-	3(7.0)	-	0.252
		CKD	1(4.0)	-	3(23.1)	0.003*
		Hypothyroidism	4(16.0)	4(9.3)	-	0.287
		IGT	-	3(7.0)	-	0.252
		NASH	2(8.0)	8(118.6)	-	0.148
Primary Outcome Variables	Weight; kg	Baseline	89.98(19.89)	96.50(20.39)	93.64(8.72)	0.406
		Follow-up	87.31(19.08)	91.80(19.40)	91.53(9.33)	0.615
	BMI; kg/m^2^	Baseline	33.27(5.56)	36.07(6.26)	34.58(5.15)	0.172
		Follow-up	32.30(5.52)	34.28(5.98)	32.65(3.43)	0.339
	HbA1c; %	Baseline	9.89(2.28)	8.02(1.89)	10.37(1.47)	0.000*
		Follow-up	7.73(2.06)	7.30(1.33)	10.48(6.87)	0.010*
Secondary Outcome Variables	FBS; mg/dL	Baseline	169.0(59.90)	172.21(65.43)	229.11(54.47)	0.183
		Follow-up	128.80(23.15)	121.71(28.96)	128.67(31.66)	0.709
	Cholesterol; mg/dL	Baseline	175.30(51.72)	165.06(35.60)	160.50(46.02)	0.580
		Follow-up	147.43(23.62)	141.67(32.41)	166.17(34.69)	0.212
	TGs; mg/dL	Baseline	209.20(154.41)	188.50(81.49)	174.17(77.66)	0.651
		Follow-up	182.23(88.14)	156.00(51.83)	153.83(50.05)	0.451
	HDL; mg/dL	Baseline	44.05(11.69)	36.26(8.09)	41.25(8.95)	0.015*
		Follow-up	42.38(12.53)	39.39(8.67)	42.33(11.25)	0.625
	LDL; mg/dL	Baseline	103.30(43.18)	100.03(34.45)	99.92(45.98)	0.952
		Follow-up	89.38(18.65)	84.19(29.46)	116.00(41.08)	0.050*
	ALT; U/L	Baseline	39.13(25.82)	49.17(40.76)	37.30(26.49)	0.519
		Follow-up	32.55(25.79)	28.35(15.02)	43.43(22.38)	0.217
	SBP; mmHg	Baseline	132.21(18.25)	135.41(18.92)	136.42(19.14)	0.749
		Follow-up	131.67(14.94)	133.20(16.72)	135.36(15.05)	0.822
	DBP; mmHg	Baseline	83.96(10.02)	83.37(8.88)	85.92(12.54)	0.734
		Follow-up	80.95(12.22)	80.94(8.65)	82.73(9.04)	0.864
	Heart rate; bpm	Baseline	89.45(9.76)	86.68(8.79)	81.42( 9.48)	0.064
		Follow-up	93.20(10.23)	90.46(9.62)	86.27(9.93)	0.218

Values are given as mean (SD) or n(%). * p<0.05 is considered significant. BMI: Body Mass Index, DL: Dyslipidemia, PCOS: Polycystic Ovary Syndrome, RA: Rheumatoid Arthritis, CKD: Chronic Kidney Disease, IGT: Impaired Glucose Tolerance, NASH: Nonalcoholic Fatty Liver Disease, OSA: Obstructive Sleep Apnoea; HbA1c: Hemoglobin A1c, FBS: Fasting Blood Sugar, TGs: Triglycerides, HDL: High-Density Lipoprotein LDL: Low-Density Lipoprotein, SBP: Systolic Blood Pressure, DBP: Diastolic Blood Pressure ALT: Alanine transaminase.

**Table-II:** Mean change in the biochemical estimations and outcome variables from baseline to follow-up visit among the patients treated with either of the GLP-1 RAs.

Variables		Baseline	Follow-up	Mean Difference	p-value

		Mean (SD)			
Primary Outcome Variables	Weight; kg	93.46(18.82)	90.34(18.01)	3.11	0.000*
	BMI; kg/m^2^	34.63(5.59)	33.40(5.52)	1.22	0.000*
	HbA1c; %	9.03(2.11)	7.95(3.26)	1.08	0.002*
Secondary Outcome Variables	FBS; mg/dL	179.45(74.74)	125.98(27.35)	53.47	0.000*
	Cholesterol; mg/dL	170.78(41.21)	145.58(30.99)	25.20	0.000*
	HDL; mg/dL	39.33(10.01)	40.96(9.91)	-1.63	0.144
	TGs; mg/dL	205.73(113.88)	161.09(62.81)	44.64	0.003*
	LDL; mg/dL	101.95(35.94)	87.66(28.63)	14.29	0.004*
	ALT; U/L	44.87(37.41)	32.49(20.28)	12.38	0.021*
	SBP; mmHg	134.95(16.70)	133.08(15.85)	1.87	0.378
	DBP; mmHg	83.76(9.54)	81.26(9.90)	2.50	0.048*
	Heart Rate; bpm	86.66(10.50)	90.62(9.93)	-3.96	0.003*

* p<0.05 was considered statistically significant.

Overall, the most common side effect reported by patients was nausea (35.8%), followed by vomiting and abdominal discomfort (Table-III). However, most patients experienced resolution within the first few days or weeks. Nevertheless, no significant difference was observed in the safety profiles of the patients treated with the various GLP-1RAs (P>.05).

**Table III:** Safety profile of obese T2D patients treated with either of the GLP-1 RAs.

Variables	Treatment Groups [n(%)]		

	Dulaglutide	Liraglutide	IDegLira

	(n=28)	(n=38)	(n=15)
Nausea	9(36.0)	18(41.9)	2(15.4)
Vomiting	6(24.0)	15(34.9)	2(15.4)
Headache	-	3(7.0)	1(7.7)
Abdominal Pain	5(20.0)	8(18.6)	3(23.1)
Diarrhea	-	1(2.3)	-
Constipation	1(4.0)	1(2.3)	-
Dizziness	1(4.0)	-	-

The treatment was discontinued in 34 patients (40.0%), 18 due to exorbitant cost, while ten patients discontinued the medicine owing to intolerable side effects, including nausea, vomiting, stomach pain, extreme weakness, fatigue, and hypoglycemia. Five patients showed resistance to consumption as they observed no advantages. One of the cases who received Dulaglutide, just four doses before discontinuation, required hospitalization due to severe, intractable vomiting, an acute kidney insult, and an increase in creatinine level up to 2.3 mg/dl.

## DISCUSSION

In the Pakistani population with T2DM, this short study demonstrates that Dulaglutide, Liraglutide alone, or combined with Degludec as add-on therapy, was effective for significant reductions in the HbA1c levels and weight.

A significant reduction in the mean HbA1c level was observed in both Dulaglutide and Liraglutide group form baseline to follow-up visit, whereas the patients treated with IDegLira showed poor glycemic control at follow-up visit in comparison to other two treatment groups. A Chinese study indicated significant decline in the HbA1c levels after treatment in both treatment and control group after 12 weeks, with comparatively better outcomes exhibited in the treatment group (Liraglutide with conventional medications) (p<0.05).^8^ The results of the Award trial and another study from Asia by Chang et al. were comparable, displaying an overall significant mean reduction in HbA1c (1.36%; p=.000).^9^,^10^ This decline in the HbA1c level was detected in all three treatment groups, although the Dulaglutide group experienced a significant reduction than the other two groups, also shown in earlier trials.^11^

Moreover, significant weight loss and BMI reduction were observed among the enrolled patients treated with GLP-1RAs. Bodyweight and BMI decreased in the three groups, though reduction differed between the groups, being maximum with Liraglutide and least with IDegLira; adding basal insulin mitigated the weight reduction with Liraglutide alone. A review from India and Madsbad reported comparable outcomes,^12^ contrary to the DUAL III trial, which revealed weight gain with IDegLira.^13^ Better control of diastolic blood pressure and cholesterol was observed, as demonstrated in a meta-analysis by Iqbal et al.^14^ Similar to earlier trials^15^,^16^, a significant improvement in ALT was seen. An increase in heart rate was observed in all three groups, which is comparable to the findings of another report by Lorenz et al.^17^ but contradicts the findings of a Pakistani study.^7^

We found no clinically significant differences between the treatment groups regarding standard safety assessments. In general, IDegLira was favorably accepted and well tolerated. In line with a previously published study,^18^ fewer participants in the IDegLira group than in the Liraglutide group experienced gastrointestinal side effects in this investigation. This is, in all probability, due to the relatively lower dose of Liraglutide administered in the fixed-dose IDegLira combination. Only one patient in the Liraglutide group experienced symptomatic hypoglycemia, but individuals taking IDegLira reported verified hypoglycemia more frequently, as reported in other research.^19^ In our study, the Liraglutide group experienced gastrointestinal adverse events more frequently than the Dulaglutide group, in comparison to another study that found identical side effect profiles.^20^

The cost was identified as the primary reason behind avoiding or discontinuing highly efficient drugs. A systematic review has also referred to this restriction on using GLP-1RAs.^21^ It is a well-known fact that these drugs work wonders in reducing diabetes-related consequences. For instance, a meta-analysis of seven studies discovered that GLP-1 RA, compared to a placebo, decreases the risk of all-cause mortality in adults with diabetes and cardiovascular disease.^22^ Except for the investigational oral Semaglutide, the formulations are currently administered as subcutaneous injections at different dosing intervals. With the introduction of oral Semaglutide and the prospect of future cost reductions, we hope to be able to provide these effective drugs to our patients.

This study has shown that GLP-1 RA, either alone or in combination with other treatments, is successful in treating obese T2D patients. It resulted in quick and sustained decrease in FPG, weight loss, and SBP, in addition to lowering HbA1c levels. South Asia lacks real-world evidence on the safety and effectiveness of GLP-1 RA, despite the global availability of such information. Small-scale studies have so far only examined the security and efficacy of Liraglutide alone in a few regions of Pakistan. Our study sought to examine Dulaglutide and IDegLira, which had not previously been examined locally. This study will provide the HCPs to have a clearer understanding and confidence while prescribing these drugs.

### Limitations:

It included the small sample size and brief follow-up period. Moreover, the study did not include other biomarkers associated with glycemic control as several other disease indicators have been recognized in association with poor glycemic control.^23^,^24^ A local study defined significant association between elevated NLR and HbA1c levels indicating poor glycemic control.^23^ In Pakistan, extensive research with a long-term monitoring period is highly recommended to provide further insight into individual case management.

## CONCLUSION

GLP-1RAs showed promising results in terms of glycemic control and weight reduction in patients with T2DM, obesity, and uncontrolled glycemia when used as adjunctive therapy without raising hypoglycemia risk. Compared to Dulaglutide, Liraglutide produced a more noticeable weight reduction, and Dulaglutide, however, demonstrated greater potential in lowering HbA1c. The GLP-1RAs used were well-tolerated, and the observed gastrointestinal adverse effects were generally mild. The main obstacle to adopting these effective medications observed in our patients was the high treatment cost.

### Authors Contribution:

**SFQ, TA:** Substantial contributions to the conception, design of the work; the acquisition, analysis, and interpretation of data for the work

**SFQ, SG, and PI:** Drafting the work or revising it critically for important intellectual content

**TA, SFQ:** Final approval of the version to be published

**SFQ, TA, SG:** Agreement to be accountable for all aspects of the work in ensuring that questions related to the accuracy or integrity of any part of the work are appropriately investigated and resolved.
